# Differential expression of islet glutaredoxin 1 and 5 with high reactive oxygen species production in a mouse model of diabesity

**DOI:** 10.1371/journal.pone.0176267

**Published:** 2017-05-24

**Authors:** Sebastian Friedrich Petry, Fatemeh Sharifpanah, Heinrich Sauer, Thomas Linn

**Affiliations:** 1 Clinical Research Unit, Center of Internal Medicine, Justus Liebig University, Giessen, Germany; 2 Department of Physiology, Faculty of Medicine, Justus Liebig University, Giessen, Germany; Baylor College of Medicine, UNITED STATES

## Abstract

The onset and progression of diabetes mellitus type 2 is highly contingent on the amount of functional beta-cell mass. An underlying cause of beta-cell decay in diabetes is oxidative stress, which markedly affects the insulin producing pancreatic cells due to their poor antioxidant defence capacity. Consequently, disturbances of cellular redox signaling have been implicated to play a major role in beta-cell loss in diabetes mellitus type 2. There is evidence suggesting that the glutaredoxin (Grx) system exerts a protective role for pancreatic islets, but the exact mechanisms have not yet been elucidated. In this study, a mouse model for diabetes mellitus type 2 was used to gain further insight into the significance of Grx for the islets of Langerhans in the diabetic metabolism. We have observed distinct differences in the expression levels of Grx in pancreatic islets between obese, diabetic db mice and lean, non-diabetic controls. This finding is the first report about a decrease of Grx expression levels in pancreatic islets of diabetic mice which was accompanied by declining insulin secretion, increase of reactive oxygen species (ROS) production level, and cell cycle alterations. These data demonstrate the essential role of the Grx system for the beta-cell during metabolic stress which may provide a new target for diabetes mellitus type 2 treatment.

## Introduction

Diabetes mellitus type 2 is hallmarked by a progressive loss of functional beta-cell mass. As from the early prediabetic stages of the disease onwards, the Islets of Langerhans suffer the detrimental effects of hyperglycaemia, free fatty acids, and inflammation [1]. As stress levels exceed the beta-cell’s coping capacity, insulin secretion fades [2, 3]. Furthermore, oxidative stress and impaired redox signaling play a pivotal role in beta-cell decay [4]. The redox regulation of cellular processes ensures cell viability and function [5]. A major actor in redox signaling and maintenance of redox balance is the glutaredoxin (Grx) system. It consists of NAPDH, glutathione, glutathione reductase and the oxidoreductase glutaredoxin. Its influence on cellular processes is based on reversible post-translational de-glutathionylation of their target’s cysteine residues. In mammals, there are four glutaredoxins, characterized as mono- or dithiol Grx depending on the number of redox-active cysteine-residuals in their active center. The dithiol Grx1 was originally found to reduce ribonucleotides and thus to ensure DNA synthesis in E. coli [6, 7]. It is mainly located in the cytoplasm, but was also reported in the intermembrane space of mitochondria and the nucleus [8, 9]. It is a major actor in the thiol-disulfide exchange and thereby involved in keeping cellular structures reduced and functional [10]. Grx1 has influence on cell differentiation [11] and regulates transcription factors, including NF-kappaB [12, 13]. NF-kappaB exerts an anti-apoptotic function in most cell types, whereas its role in the beta-cell is dependent on its activators. A protective effect against apoptosis induced by TNF-alpha is suggested [14]. Furthermore, researchers demonstrated that Grx1 promotes insulin secretion in MIN6 cells and isolated rat islets [15] while Grx1 knockout resulted in impaired insulin secretion [16]. The second dithiol, Grx2, forms iron-sulfur clusters which act as a redox sensor [17, 18]. As a regulatory mechanism of the redox state in the mitochondria [19] it has protective effects from apoptosis [20]. The monothiol Grx3 can form iron-sulfur clusters [21] as well and is necessary for haem synthesis [22]. It has protective and immunomodulatory effects, too [23, 24]. Grx5 was studied in yeast mutants lacking the enzyme. These featured increased susceptibility to oxidative and osmotic stress. Elevated ROS production, accumulation of iron and inactivation of enzymes requiring Fe-S clusters were noted [25, 26]. These affected enzymes are not only required for glucose processing and thereby insulin secretion, but iron accumulation is also known to induce secondary complications in diabetes [27]. Due to their diverse functions and their substrate specifity, alterations in glutaredoxin activity and expression can have massive impact on cellular pathways. Therefore, a key role in diabetes has been implicated [28]. The metabolically highly active beta-cell suffers from low antioxidant capacity. Several enzymes, including superoxide dismutase, catalase and glutathione peroxidase were shown to be expressed less in mouse islets in comparison to other tissues [29]. Oxidative stress disrupts the physiology of insulin secretion at several stages. GLUT2 (Glucose transporter 2) expression is reduced in rodent models for diabesity [30, 31] and mitochondrial dysfunction [32–34] was reported. Furthermore, oxidative modifications of ATP-sensitive potassium channels as well as hyperpolarization of the cell membrane conducted by ROS can both take influence on insulin secretion [35, 36]. Surprisingly, there are few data regarding glutaredoxins in the islets of diabetic mice. We hypothesized that they play a role in the challenged beta-cell during the onset and progression of diabetes mellitus type 2. Therefore, we screened islets of diabetic db/db mice in comparison to lean db/+ littermates. Homozygote db mice are marked by total leptin resistance due to a receptor mutation [37] and thus develop obesity and diabetes (“diabesity”) [38]. We found lower expression of all four glutaredoxins in diabetic mice in comparison to lean controls. The effect was most pronounced for Grx1 and 5, which lead us to further investigate both redoxins. For the first time we detected distinguished differences in Grx expression in the islets of Langerhans in a mouse model for diabetes mellitus type 2 which could be correlated to insulin expression, cell cycle, and ROS production.

## Materials and methods

### Ethics statement

Animal research was approved by and conducted in accordance with institutional animal welfare officer, Chair of Animal Welfare of the Justus Liebig University Giessen, and Regional Administrative Council Giessen, Veterinary Department, under the code GI20/11-Nr.A18/2010. 3Rs were applied for reducing the number of required mice and reduce potential suffering, enrichment was applied to the IVC. Scoring was done daily. Mice were anaesthetized with ketamin / xylazin i.p. and bled to death by incision of the abdominal aorta before removing the pancreas and other organs. The ARRIVE Guidelines Checklist (Animal Research: Reporting In Vivo Experiments) is available in the Supporting information section (S1 File).

### Animal model

40 male BKS(D)-Leprdb/JOrlRj (db/db) mice and 40 BKS(D)-Leprdb/JOrlRj Témoin (db/+) control mice aged 5 weeks were bought from Charles River (Sulzfeld, Germany) and given one week to adapt to the animal facility. Number of required mice was calculated regarding manifestation rate of diabetes according to literature with type I error of 0.05 and type II error of 0.2. Animals were housed according to institutional guidelines (room temperature 22 ± 0.5°C, 12 hours light / dark cycle, 60% humidity) with tap water and standard diet pellet food (Altromin, Lage, Germany) ad libitum in individually ventilated cages in groups of five mice. Mice were observed from 6 to 18 weeks of age. Blood glucose (Glucometer OneTouch Ultra 2, LifeScan, Neckargemünd, Germany) and body weight were measured weekly. Blood for glucose measurement was collected by puncturing the tip of the tail on conscious animals after overnight fasting. Pancreatectomy for IHC and islet isolation for qRT-PCR were done at 6, 12 and 18 weeks of age. In order to harvest pancreata mice were sedated by narcotic agent (1 ml Ketamine 10%, 0.8 ml Xylazine 2%, 8.2 ml NaCl 0.9%; 0.1 ml / 10 g body weight). Next, the abdominal wall was excised to expose the aorta which was cut to drain the blood before the organ was removed.

### Islet isolation

Islets were isolated as described before by our department [39]. Briefly, the extracted pancreas was perfused with 4 mg / ml collagenase B (Roche, Mannheim, Germany) dissolved in 1% Hank’s solution (Biochrom, Berlin, Germany) supplemented with 35 ml Hepes buffer (Biochrom, Berlin, Germany), 10 ml Ciprofloxacin, 10 ml Penicillin-Streptomycin, and 1 ml Gentamycin through the ductus pancreaticus. The perfused pancreas was mechanically chopped with scissors before 10 minutes of incubation in collagenase solution at 37°C in a shaking water bath. After every 3 minutes of collagenase digestion the sample was vortexed for 10 seconds. The digested tissue was shaken by hand for two more minutes and the digestion process was eventually stopped by placing the tube containing the tissue on ice and adding cold Hank’s solution. Following 3 minutes of centrifugation at 1500 rpm, the supernatant was discarded and the pellet was dissolved in 15% P/FCS dissolved in Medium 199 (Gibco, Karlsruhe, Germany) and fetal bovine serum (biowest, Nuaillé, France) at room temperature. The islets were hand-picked under stereomicroscope and incubated overnight at 37°C to overcome the isolation stress.

### Immunohistochemistry

IHC was used for detection of insulin (Dako, Hamburg, Germany), Grx1 (Santa Cruz Biotechnology, USA) and 5 (kindy provided by Prof. Lillig / Dr. Hanschmann as described in [40]), Ki-67 (Dako, Hamburg, Germany) as a marker for proliferation and activated caspase-3 (Cell Signaling, Frankfurt, Germany) as a marker for apoptosis. Pancreata were fixed with Zamboni (paraformaldehyde in picric acid and PBS as described in [41]) for four hours, then washed and stored in PBS supplemented with 18% sucrose solution overnight. Organs were embedded in cryoblock embedding medium (Biosystems, Nunningen, Switzerland) and frozen at -80°C. Sectioning was done using Leica Crysostat CM1850 (Leica, Wetzlar, Germany). Slides were washed with PBS and blocked with 1% donkey serum dissolved in PBS containing 0.3% Triton X-100 (0.3% PBST) for 20 minutes. For Ki-67 staining, antigen retrieval was performed with NaOH 0.09 M for three minutes followed by another wash cycle. Sections were incubated with primary antibodies diluted in 1% donkey serum dissolved in 0.3% PBST overnight at 4°C. Secondary antibodies in 5% mouse serum were applied for one hour at room temperature. Nuclei were stained with Hoechst (Calbiochem, Darmstadt, Germany) in 0.1% TRIS buffer pH 7.6 and samples were preserved with ProLong Gold (Invitrogen, Karlsruhe, Germany). Pancreata were entirely sectioned. Sections were assessed for their quality, i.e. structurally damaged ones were not used. Two consecutive sections were regarded as one (results were divided by two for analysis). An interval of 140 *μ*m was maintained between these pairs of slides which were included in the analysis in order to avoid multiple inclusion of islets. When islet area exceeded 140 *μ*m ² in 12 weeks old db/db mice, double inclusion was carefully avoided by manual comparison of slides. 12 slides per pancreas, including 24 to 384 islets per organ, were observed. Total islet count included in the study was 2221 with 123 islets per mouse on average. Islet count was consistent with the number of islets isolated during islet isolation. Slides were prepared simultaneously in batches during immunohistology for appropriate comparability. For qualitative assessment of redoxin staining patterns in islets, immunostained slides were screened for islets. Images were taken, background staining was removed, and images were converted to gray scale for more effective and accurate comparison. Representative images were then selected manually.

### Measurement of islet area and protein expression level

Size of islet area as well as protein expression level of Grx1, Grx5 and insulin were measured by using custom scripts for ImageJ (Wayne Rasband, National Institutes of Health, USA). Images were taken with Leica Application Suite v 3.8.0 using digital microscope camera DFC 420 C (Leica, Wetzlar, Germany) as described before [42]. Software was calibrated to match the images’ scale and mean islet area was obtained. For analysis of protein expression level of insulin, Grx1 and 5, gray scaled pictures taken from a batch of simultaneously prepared stainings were normalized by removing background using slides without primary antibodies and calculating mean fluorescent intensity in islets. This resulted in mean grey values ranging from 0 (0%) to 255 (100%). In order to quantify redoxin staining area, the area stained by the antibody against the respective redoxin was set into relation with the area stained by insulin.

### RNA isolation, cDNA synthesis and qRT-PCR

Extraction of RNA from harvested islets was done using RNeasy Plus Micro Kit (Qiagen, Düsseldorf, Germany). Total RNA concentration was determined by OD260 nm method using NanoDrop 1000 spectrophotometer (Thermo Scientific, Schwerte, Germany). cDNA was synthesised with SuperScript III Reverse Transcriptase kit (Invitrogen, Darmstadt, Germany). qRT-PR was carried out on Real-Time PCR System StepOnePlus (Applied Biosystems). Each PCR consisted of 10 min denaturation at 95°C, followed by 40 cycles of denaturation (95°C, 10 s) and annealing / extension (60°C, 1 min). Primer concentration for qRT-PCR was 20 pM. Primer (Invitrogen, Darmstadt, Germany) sequences were as follows:

*beta-actin* (housekeeping):

fwd GTG GGA ATG GGT CAG AAG G, rev GAG GCA TAC AGG GAC AGC A;

*INS1*:

fwd TAT AAA GCT GGT GGG CAT CC, rev GGG ACC ACA AAG ATG CTG TT;

*Grx1*:

fwd GAG CAG TTG GAC GCG CTG G, rev CTC GCC ATT GAG GTA CAC TTG C;

*Grx5*:

fwd GAA GAA GGA CAA GGT GGT GGT CTT C, rev GCA TCT GCA GAA GAA TGT CAC AGC

Relative mRNA expression values were obtained by normalizing CT values of the target genes in comparison with CT values of the housekeeping gene using the delta-CT method.

### Measurement of ROS production

Intracellular reactive oxygen species (ROS) production level in isolated islets were detected using 2’,7’-dichlorofluorescein diacetate (DCFH-DA) indicator dye (Sigma, Munich, Germany). DCFH-DA is a non-fluorescent, cell-permeable compound which is cleaved by intracellular esterases to 2’,7’-dichlorofluorescein (DCFH) and thereby trapped within cells. DCFH is membrane impermeable and a variety of intracellular ROS rapidly oxidize it to the highly fluorescent DCF (2’,7’-dichloro-fluorescein) [43, 44]. Isolated islet cells of 24 weeks old animals were categorized in three different groups as untreated control, high glucose treated (20 mM for 2 hours) and TNF-alpha treated (1*μ*M for 15 minutes). The above mentioned samples were incubated in serum-free medium containing 10*μ*M DCFH-DA indicator dye dissolved in dimethylsulfoxide (DMSO) (Sigma, Munich, Germany). After 30 min incubation at 37°C in the dark, samples were rinsed with pre-warmed serum-free medium and immediately analyzed with confocal laser scanning microscope. Intracellular DCF fluorescence (corrected for background fluorescence) was evaluated in 3600 *μ*m^2^ regions of interest using an overlay mask unless otherwise indicated. For fluorescence excitation, the 488 nm band of the argon ion laser of a confocal laser scanning microscope (Leica SP2 AOBS, Bensheim, Germany) was used. Emission was recorded using a longpass LP515 nm filter set. All islet cells per condition were photographed and fluorescence intensities were quantified with Leica Simulator software.

### Statistical analysis

Statistical analysis was performed using Graph Pad Prism 5 (GraphPad Software, San Diego, USA) using Mann Whitney test and two-way Anova as appropriate. Data are given as mean values ± SEM, with n denoting the number of experiments unless otherwise indicated. A p-value < 0.05 was considered significant.

## Results

### A phenotype of diabesity in rodents

**Fig 1:**
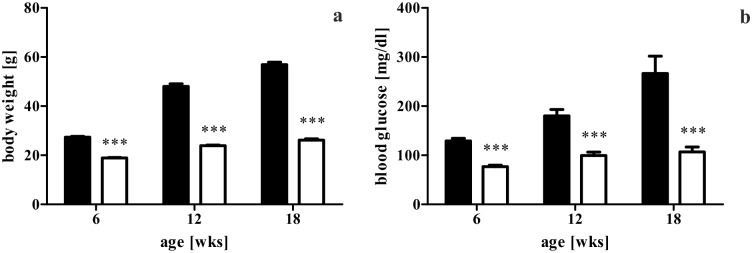
Body weight and fasting blood glucose level of db/db and db/+ mice. (a) Body weight of db/db and db/+ mice. (b) Fasting blood glucose levels of db/db and db/+ mice. Data depicted from 6, 12, and 18 weeks of age, corresponding to pancreatectomy. Values are mean ± SEM (n = 11-40 mice), black bars represent db/db mice, white bars represent db/+ mice, *** denotes *p* < 0.0001.

The leptin-resistant db mouse was employed for investigation of glutaredoxins in diabetes. It is known that homozygous db mice exhibit a distinct aspect of diabesity. Consecutively, a strong obese and diabetic phenotype was developed during the 12 weeks study period. Homozygous animals constantly showed a significantly higher body weight as well as fasting blood glucose during the observation period from 6 to 18 weeks of age (*p* < 0.0001). At the end of the observation period, homozygotes with 57 g on average were twice as heavy as heterozygotes with 26 g (Fig 1a). Their fasting blood glucose exceeded 200 mg/dl from 13 weeks of age onwards while heterozygotes showed no significant increase from 12 to 18 weeks of age (Fig 1b).

### Pathohistomorphic changes with distinct morphology and high cell turnover

**Fig 2:**
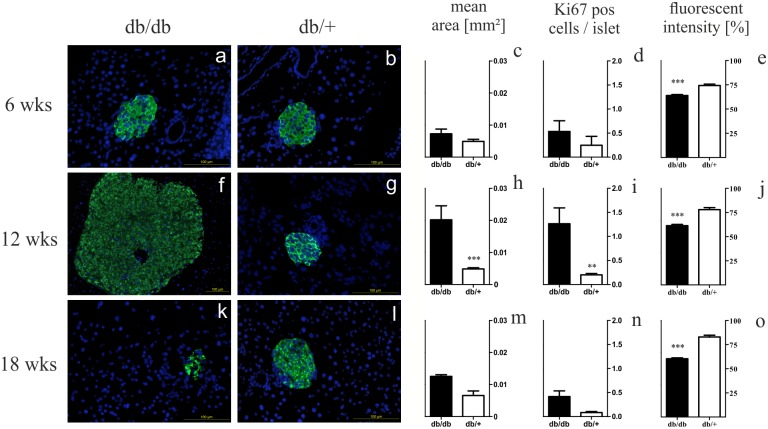
Morphology and quantification of area, proliferation rate, and fluorescent intensity of db/db and db/+ islets. (a, b, f, g, k, l) Representative images taken of immunostained islets at 6, 12 and 18 weeks are shown in comparison (green: insulin, blue: nuclei; bars indicate 100 *μ*m^2^). (c, h, m) Quantification of mean islet area as measured with ImageJ corresponding to insulin staining area. (d, i, n) Proliferation of islet cells analyzed by Ki-67 positive cells per islet. (e, j, o) Semiquantitative analysis of insulin staining. Black bars represent db/db mice, white bars represent db/+ mice, n = 3 mice, *** denotes *p* < 0.001, ** denotes *p* < 0.005.

After having ensured a strong phenotype in the selected strain of db mice as well as the suitability of heterozygous animals as controls, the histology of their pancreatic islets was examined. Assessment of islet count during islet isolation revealed more islets in db/db animals at all time points (*p* < 0.001 at 6 weeks of age, *p* < 0.05 on average, data not shown). In both groups, lowest pancreatic islet count was observed at the age of 12 weeks. Morphological studies by immunohistology exposed larger islets occuring together with fragmented small ones in homozygotes (Fig 2). At the age of 6 weeks islets of homozygotes were already enlarged but shaped normally (Fig 2a). Larger islets in 12 weeks old db/db mice resulted in vast islet formations (Fig 2f). As homozygotes grew older, small abnormally shaped islets increased in number (Fig 2k). Changes in islets of heterozygotes were more moderate (Fig 2b, 2g and 2l). These findings were quantified by measuring islet area in immunostained slides. We found larger mean islet area in db/db animals at all time points with a maximum at 12 weeks (*p* < 0.001 at 12 weeks and *p* < 0.005 on average) (Fig 2c, 2h and 2m). On average, db/db mice had 2.5-fold larger islets. Respectively, islet proliferation was measured by counting of cells positive for Ki-67 staining. We found higher proliferation of db/db islets (*p* < 0.005 at 12 weeks and on average) (Fig 2d, 2i and 2n). Again, a peak was found at 12 weeks in homozygotes, while proliferation decreased in ageing animals of both groups. On average, homozygotes featured a four-fold higher proliferation rate. Slides were also stained against activated caspase-3 and analysed qualitatively. Apoptosis was higher in 6 and 12 weeks old homozygotes in comparison to heterozygotes (data not shown).

### Receding insulin content in the islets of Langerhans

**Fig 3:**
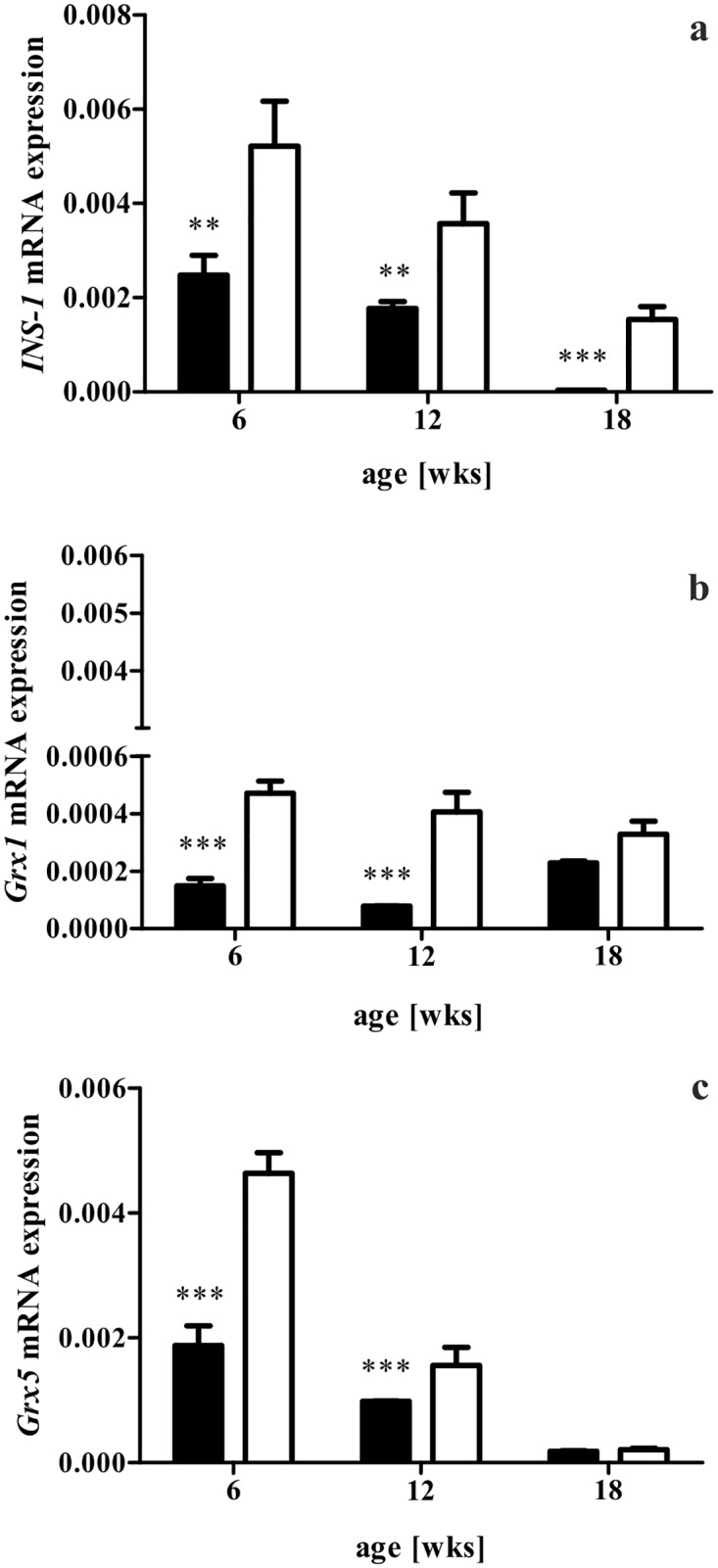
Gene expression of *INS1*, *Grx1*, and *Grx5* in db/db and db/+ islets. Gene expression was evaluated by qRT-PCR. (a) *INS1* expression declined in both groups of mice in relation to their age, but controls exhibited significantly higher expression levels at all time points. (b) *Grx1* expression was higher in db/+ mice at all time points. A slight decrease in controls was observed, while db/db animals featured a gap at 12 weeks of age. (c) *Grx5* expression decreased in both groups with age with higher levels in db/+ islets. Values are mean ± SEM (n = 4—6 mice) and normalized with beta-actin, black bars represent db/db mice, white bars represent db/+ mice, *** denotes *p* < 0.0001, ** denotes *p* < 0.005.

Both the phenotype of diabesity as well as the marked histological alterations in db/db mice were accompanied by a depletion of insulin content in islets, as our respective analysis revealed. This loss correlated to the extent of body weight and blood glucose levels of db/db animals. Quantification of insulin staining of immunohistology confirmed the impression of lower insulin content in db/db islets when compared to db/+ specimen (*p* < 0.0001) (Fig 2e, 2j and 2o). Regarding the genetic level, homozygotes showed a 70-fold drop in *INS1* expression from 6 to 18 weeks of age (*p* < 0.0001) (Fig 3a). In comparison to heterozygotes, *INS1* expression was twice as low at 6 and 12 weeks of age (*p* < 0.05) and 45-fold lower at 18 weeks (*p* < 0.0001). Heterozygote expression was declining less markedly.

### Marked differences in islet redoxin expression

**Fig 4:**
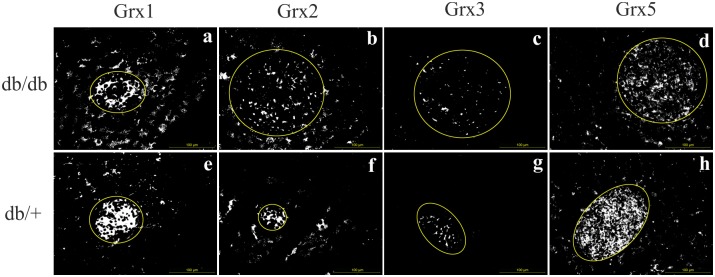
Qualitative comparison of the Grx system in db/db and db/+ islets. Representative monochrome pictures of Grx staining pattern captured of islets of 12 weeks old db/db and db/+ mice. (a, e) Grx1, (b, f) Grx2, (c, g) Grx3, (d, h) Grx5. Staining patterns suggested higher expression in db/+ mice. The difference was most pronounced for Grx1 and 5. 200x, yellow circles indicate islets, bars indicate 100 *μ*m^2^.

Our aim was to correlate the witnessed differences between obese, diabetic and lean, non-diabetic mice with changes in glutaredoxin expression. For screening, we assessed the pattern as well as fluorescent intensity of immunohistologically stained islets from 12 weeks old mice of both groups. This timepoint appeared most promising for the observed marked differences in shape, insulin content and proliferation detected in our previous analysis. We discovered visible differences with more dense and extense staining in db/+ animals for all four glutaredoxins, which were most pronounced for Grx1 and 5 (Fig 4).

### Reduced glutaredoxin 1 and 5 levels in islets during diabesity

**Fig 5:**
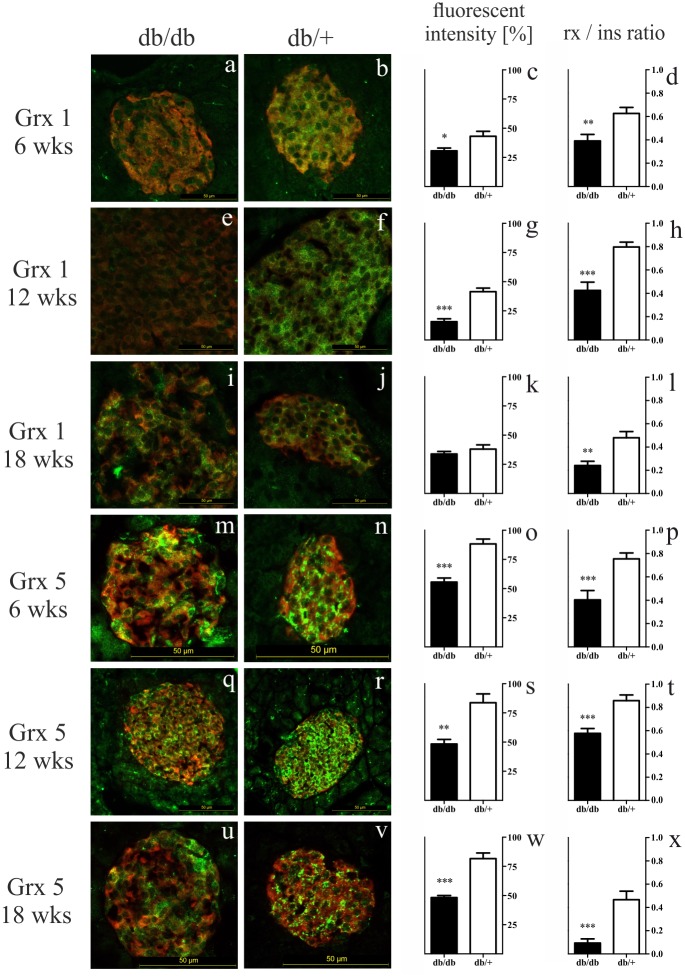
Representative images of Grx1 and 5 staining, quantification of Grx to insulin ratio, and fluorescent intensity of db/db and db/+ islets. (a, b, e, f, i, j, m, n, q, r, u, v) Representative images taken of immunostained islets at 6, 12 and 18 weeks are shown in comparison (green: Grx1 / 5, red: insulin, bars indicate 50 *μ*m^2^). (c, g, k, o, s, w) Semiquantitative analysis of Grx1 / 5 staining. (d, h, l, p, t, x) Quantification of Grx 1 / 5 to insulin staining ratio. Black bars represent db/db mice, white bars represent db/+ mice, n = 3 mice, *** denotes *p* < 0.0001, ** denotes *p* < 0.005, * denotes *p* < 0.05.

Based on the screening of glutaredoxins in islets, we carried out further analysis for Grx 1 and 5. Staining in db/db islets was more scarce and extended over a smaller area when compared to control islets. Furthermore, Grx5 staining patterns were more intense and more extensive than Grx1 patterns. Findings were confirmed by semiquantitative analysis of Grx fluorescent intensity as well as quantification of Grx to insulin staining ratio (Fig 5). Control islets featured higher Grx fluorescent intensity and Grx to insulin ratio for both redoxins and all time points. Grx5 was the redoxin with higher expression. To confirm the findings on the genome level, gene expression of *Grx1* and *5* was analysed by qRT-PCR in isolated islets. Corresponding to higher fluorescent intensity and area, heterozygotes showed higher gene expression (*p* < 0.0001 at 6 and 12 weeks) for *Grx1* at all time points (Fig 3b). In homozygote islets, a nadir was found at 12 weeks, while expression im heterozygote islets was stable. Analysis of *Grx5* expression revealed a significant decrease (*p* < 0.0001) in both db/db and db/+ animals with controls exhibiting higher expression at all time points (*p* < 0.0001 at 6 and 12 weeks) (Fig 3c). qRT-PCR revealed a markedly higher expression of *Grx5* when compared to *Grx1*.

### Enhanced ROS production in Grx-deficient islets

**Fig 6:**
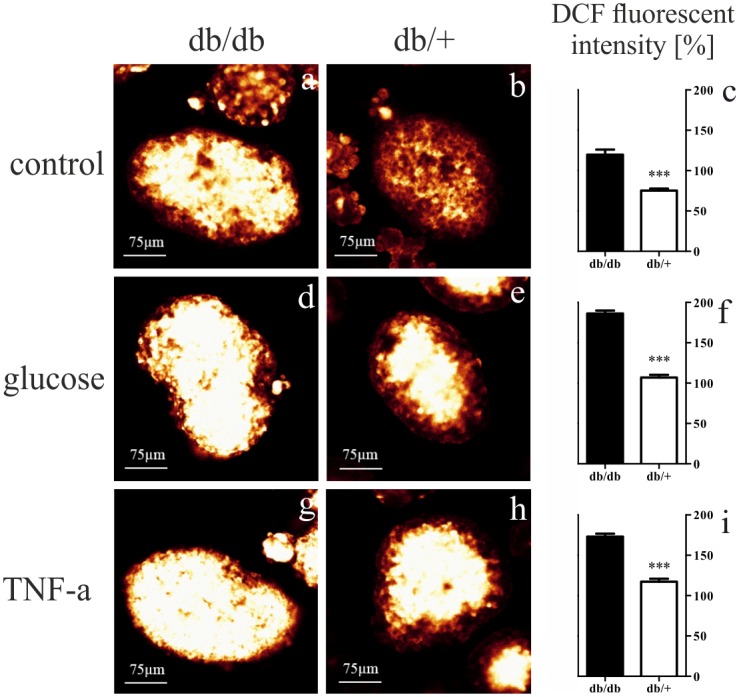
Representative images of ROS measurements and quantification in islets of db/db and db/+ mice. (a, b, d, e, g, h) Representative images show DCF stained pancreatic islets without any treatment as well as upon treatment with either glucose or TNF-alpha (bars represent 75 *μ*m). (c, f, i) Quantification of DCT fluorescence intensity revealed significantly higher ROS production in db/db islets with a more pronounced rise after exposure to high glucose and TNF-alpha treatment in comparison to db/+ islets. Values are mean ± SEM (n = 54—139 islets), black bars represent db/db islets, white bars represent db/+ islets, *** denotes *p* < 0.0001.

In order to further evaluate the significance of Grx-deficiency in mouse islets in diabetes, we analyzed ROS production level in isolated pancreatic islets using DCF staining. It was evaluated in untreated islets as well as islets pretreated with high glucose and TNF-alpha in order to stimulate hyperglycaemic and inflammatory stress as apparent during diabetes mellitus type 2. Isolated pancreatic islets from homozygous mice indicated higher ROS production level without any stimulation as well as upon treatment with either glucose or TNF-alpha compared to heterozygotes (*p* < 0.0001). Both groups showed an elevation in ROS content upon treatment with either glucose or TNF-alpha, but with a markedly higher level in db/db islets (Fig 6).

## Discussion

The course of diabetes mellitus type 2 is highly dependent on the preservation of a healthy beta-cell mass and insulin secretion capacity, which both are notably impaired by oxidative stress and disturbed redox signaling [45]. It was previously shown that the Grx system has positive impact on insulin secretion [16, 46, 47]. Furthermore, tissue-specific expression of Grx was reported [48]. Plasmatic Grx activity differs between healthy subjects and patients suffering from diabetes mellitus type 2 [49]. Evidence for a protective role of the Grx system in the diabetic metabolism has arisen [50–54], but its exact significance for the beta-cell is still unknown. Thus, the aim of this study was to evaluate the relevance of the glutathione-dependent oxidoreductase system as a potential protection machinery in an in vitro model for diabetes mellitus type 2. A high level of oxidative phosphorylation is mandatory for the metabolically active beta-cell, especially when challenged by metabolic stress. As a result, mitochondrial ROS are considered a requirement for unimpaired insulin secretion [55, 56]. However, it is accepted that an abundance of ROS induces oxidative stress, exerts detrimental effects on cellular structures and proteins, and disturbs redox signaling. Accordingly, permanent excess of ROS is seen as noxious to insulin secretion [57]. Elevated ROS production in islets of diabetic rodents were confirmed by others [58], and overexpression of radical-scavenging enzymes resulted in protection of ROS-mediated induction of diabetes [59]. The beta-cell is especially prone to oxidative damage for its low expression of enzymes such as catalase and glutathione peroxidase, which are mainly involved in detoxification of H_2_O_2_ [29]. The glutaredoxin system has been proven to be expressed in mice islets [48] and may represent an important alternative pathway in detoxification of ROS for the beta-cell. The four mammalian glutaredoxins are located in the major cellular compartments [8, 21, 26, 60] and are thereby involved in a broad range of functions. Thus, it is acknowledged that they feature varying expression patterns and activity when the beta-cell is challenged by metabolic stress. In the present study, we identified significant differences in Grx expression between diabese mice and lean controls for all four oxidoreductases, which were most pronounced for the mainly cytoplasmic Grx1 as well as the mitochondrial Grx5. Our data indicate that Grx1 expression correlated negatively with average size of pancreatic islets as well as proliferation rate, and positively with islet count in homozygotes. Grx1 protein and mRNA expression were elevated at 6 and 18 weeks when beta-cell turnover was at its minimum. At these time points homozygous mice featured high islet count and small islets with low proliferation rate. By contrast, we found reduced mRNA and protein expression of Grx1 at 12 weeks when islet count was low, but islets were large and showed elevated proliferation. Furthermore, a correlation was found between higher and stable expression in heterozygotes and less apoptosis of islet cells, elevated insulin expression and stable blood glucose levels when compared to homozygotes. These observations support the anti-apoptotic [61, 62] and pro-proliferative role [11] of Grx1. Grx1 is a major catalyst of post-translational modification of proteins via de-glutathionylation [61, 63], reversing detrimental glutathionylation by ROS. Among its targets are NF-kappaB, a key regulator of apoptosis [64], and PKC-alpha [65]. Previous studies suggest a positive impact of PKC-alpha on insulin secretion via maintenance of calcium channels [66, 67]. Further, Grx1 restores aldose reductase [68], an enzyme which is required for glucose processing if hexokinase is saturated due to glucose overload. A recent study linked Grx to adenosine monophosphate-activated protein kinase (AMPK) activation and thereby stabilization of insulin secretion [54]. Consequently, Grx1 might support generation of new islets as well as growth of pre-existing islets, and further sustain islet metabolic activity in attempt to maintain glucose homeostasis and preserve insulin secretion capacity. In this study, Grx5 featured a significant reduction in both groups of mice, which was markedly more pronounced in db/db animals and correlated with fading insulin expression and rising blood glucose levels. The Grx5 enzyme is an important actor in composition of iron-sulfur clusters in the mitochondria [26]. Therefore, it is essential for a broad range of enzymes relying on these clusters, among which numerous have relevance for the respiratory chain [26]. Thus, the connection between Grx5 and oxidative phosphorylation might explain the co-occurrence of reduced insulin as well as Grx5 expression in this study. It has been reported that Grx5-deficiency increases the susceptibility to oxidative stress [25]. Furthermore, a lack of Grx5 enzyme was also correlated to cellular iron overload [69]. Iron is known to catalyze ROS production and thereby mediate apoptosis [70]. The link between metabolic stress and iron metabolism was shown in an in vivo model for diabetes mellitus type 2 with defective iron channels which featured stronger beta-cell viability [71]. Also, iron chelator treatment and dietary iron restriction had beneficial effects on glucose homeostasis in rodents [72]. Therefore, Grx5 might play a key role in maintaining mitochondrial functionality and prevent detrimental impact of iron accumulation. At the present time, the exact significance of reduced Grx1 and 5 levels in diabese db/db mice remains to be studied. Functional experiments will elucidate whether reduced redoxin levels are cause or result of impaired insulin secretion in islets of diabese animals. Regulators and effectors of islet redoxins have to be identified and modulation of redoxin expression should be investigated regarding its influence on islet viability and metabolic activity. Further, immunohistological analysis in this study was limited by a low number of mice (n = 3). However, an appropriate amount of islets per animal was used for analysis and results were consistent among animals. Moreover, genome analysis via mRNA expression was carried out in 4 to 6 mice per timepoint and results were consistent with protein analysis.

## Conclusion

**Fig 7:**
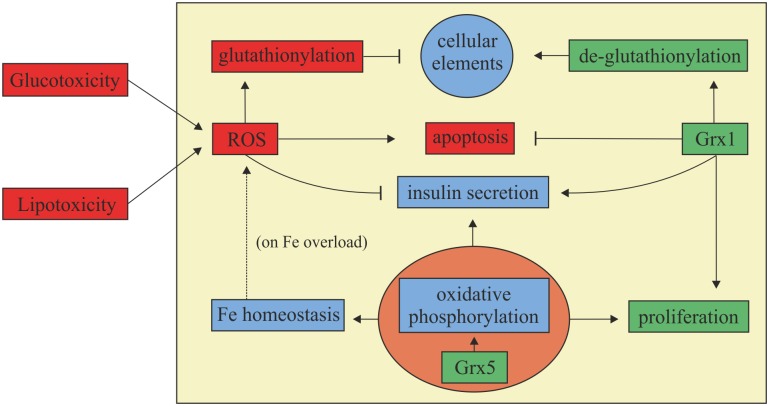
Summary. Both gluco- and lipotoxicity are extracellular promoters of ROS generation. ROS are harmful to cellular elements as they catalyze their glutathionylation. When the cell’s antioxidant capacity is depleted, cell death occurs. Regarding the beta-cell, ROS impair insulin secretion. Grx1 and 5 wield protective properties. Grx1 is a major actor in de-glutathionylation, thereby reversing the harmful effects of ROS on its targets, exerting anti-apoptotic and pro-proliferative effects, and preserving insulin secretion. Grx5 has impact on the respiratory chain and cellular iron homeostasis by transferring iron-sulfur clusters to respective apoproteins. Hence, it supports cell viability and function, allows proliferation and counteracts iron accumulation which would promote ROS formation.

In conclusion, our findings demonstrate a correlation between glutaredoxins and dysfunction of the islets of Langerhans in a mouse model for diabesity, which has not been described before. We propose that deficiency of Grx1 and 5 is connected to impaired insulin secretion and beta-cell decay in diabetes mellitus type 2. A summary of the obeserved correlations in reference to literature is given in Fig 7.

## Supporting information

S1 File The ARRIVE guidelines checklist. The ARRIVE Guidelines Checklist (Animal Research: Reporting In Vivo Experiments) was followed and is available.
(PDF)
